# Updated Diagnostic Criteria for Paraneoplastic Neurologic Syndromes

**DOI:** 10.1212/NXI.0000000000001014

**Published:** 2021-05-18

**Authors:** Francesc Graus, Alberto Vogrig, Sergio Muñiz-Castrillo, Jean-Christophe G. Antoine, Virginie Desestret, Divyanshu Dubey, Bruno Giometto, Sarosh R. Irani, Bastien Joubert, Frank Leypoldt, Andrew McKeon, Harald Prüss, Dimitri Psimaras, Laure Thomas, Maarten J. Titulaer, Christian A. Vedeler, Jan J. Verschuuren, Josep Dalmau, Jerome Honnorat

**Affiliations:** From the Neuroimmunology Program (F.G., J.D.), Institut d'Investigacions Biomèdiques August Pi i Sunyer (IDIBAPS), Barcelona, Spain; Centre de Référence National pour les Syndromes Neurologiques Paranéoplasique (A.V., S.M.-C., J.-C.G.A., V.D., B.J., L.T., J.H.), Hôpital Neurologique, Hospices Civils de Lyon; SynatAc Team (A.V., S.M.-C., V.D., B.J., L.T., J.H.), NeuroMyoGene Institute, INSERM U1217/CNRS UMR5310, Lyon; Université Claude Bernard Lyon 1 (A.V., S.M.-C., V.D., B.J., L.T., J.H.), Université de Lyon; Service de Neurologie (J.-C.G.A.), CHU de Saint-Etienne, France; Department of Neurology (D.D., A.M.), Laboratory Medicine and Pathology, Mayo Clinic, Rochester, MN; Neurology Unit (B.G.), Trento Hospital, Azienda Provinciale per I Servizi Sanitari (APSS) di Trento, Italy; Oxford Autoimmune Neurology Group (S.R.I.), Nuffield Department of Clinical Neurosciences, John Radcliffe Hospital, University of Oxford, United Kingdom; Neuroimmunology Section (F.L.), Institute of Clinical Chemistry, University Hospital Schleswig-Holstein Kiel/Lübeck; German Center for Neurodegenerative Diseases (DZNE) Berlin (H.P.), and Department of Neurology and Experimental Neurology (H.P.), Charité–Universitätsmedizin Berlin, Germany; Centre de Compétence des Syndromes Neurologiques Paranéoplasiques et Encéphalites Autoimmunes (D.P.), Groupe Hospitalier Pitié-Salpêtrière, Paris, France; Department of Neurology 2 Mazarin (D.P.), and INSERM U 1127 (D.P.), CNRS UMR 7225, Centre de Recherche de l'Institut du Cerveau et de la Moelle Epinière Groupe, Hospitalier Pitié-Salpêtriêre et Université Pierre et Marie Curie-Paris 6, AP-HP, France; Department of Neurology (M.J.T.), Erasmus MC University Medical Center, Rotterdam, the Netherlands; Department of Clinical Medicine (C.A.V.), University of Bergen; Department of Neurology (C.A.V.), Haukeland University Hospital; Neuro-SysMed–Centre of Excellence for Experimental Therapy in Neurology (C.A.V.), Departments of Neurology and Clinical Medicine, Bergen, Norway; and Neurology Department (J.J.V.), Leiden University Medical Center, the Netherlands.

## Abstract

**Objective:**

The contemporary diagnosis of paraneoplastic neurologic syndromes (PNSs) requires an increasing understanding of their clinical, immunologic, and oncologic heterogeneity. The 2004 PNS criteria are partially outdated due to advances in PNS research in the last 16 years leading to the identification of new phenotypes and antibodies that have transformed the diagnostic approach to PNS. Here, we propose updated diagnostic criteria for PNS.

**Methods:**

A panel of experts developed by consensus a modified set of diagnostic PNS criteria for clinical decision making and research purposes. The panel reappraised the 2004 criteria alongside new knowledge on PNS obtained from published and unpublished data generated by the different laboratories involved in the project.

**Results:**

The panel proposed to substitute “classical syndromes” with the term “high-risk phenotypes” for cancer and introduce the concept of “intermediate-risk phenotypes.” The term “onconeural antibody” was replaced by “high risk” (>70% associated with cancer) and “intermediate risk” (30%–70% associated with cancer) antibodies. The panel classified 3 levels of evidence for PNS: definite, probable, and possible. Each level can be reached by using the PNS-Care Score, which combines clinical phenotype, antibody type, the presence or absence of cancer, and time of follow-up. With the exception of opsoclonus-myoclonus, the diagnosis of definite PNS requires the presence of high- or intermediate-risk antibodies. Specific recommendations for similar syndromes triggered by immune checkpoint inhibitors are also provided.

**Conclusions:**

The proposed criteria and recommendations should be used to enhance the clinical care of patients with PNS and to encourage standardization of research initiatives addressing PNS.

Paraneoplastic neurologic syndromes (PNSs) are remote effects of cancer with an immune-mediated pathogenesis.^1,2^ The diagnosis of PNS can be difficult and requires careful exclusion of direct involvement of the nervous system by cancer, such as brain metastasis or carcinomatous meningitis, and indirect involvement caused by coagulopathy, treatment-related neurotoxicity, metabolic problems, or infections.^1,3^ PNSs develop in approximately 1 of 300 patients with cancer.^3^ Few population-based epidemiologic studies have been performed in the field of PNS. Yet, stated incidence varies from 1.6 to 8.9 per million person-years, suggesting that underdiagnosis and underreporting are still relevant issues.^3,4^ It is likely that the expanding use of immune checkpoint inhibitors (ICIs) in oncologic practice will lead to an increased frequency of similar syndromes.^5,6^

In 2004, a set of recommended diagnostic criteria for PNS were defined by a panel of international experts and became the standard for clinical and research purposes.^7^ Since then, several advances in the field of PNS suggest that this is an opportune moment to update the 2004 criteria: first, the characterization of new intraneuronal proteins as targets of autoantibodies in PNS; second, the discovery of pathogenic antibodies against neuronal surface antigens in neurologic syndromes that can occur with or without cancer, a group which emphasizes the need for a new definition of an onconeural antibody; and third, some of the 2004 fundamental criteria needed to be redefined or modified. For example, the definition of definite PNS solely based on the presence of onconeural antibodies is no longer adequate. Similarly, in the elderly population, where the prevalence of some tumors is high (e.g., prostate cancer), the use of criteria that rely on generic tumor association may overestimate the real burden of PNS.

In September 2019, a group of international experts (PNS-Care panel) was convened and charged with revising the diagnostic criteria of PNS to benefit clinical decision making, epidemiologic, and research purposes and to address the ancillary issues outlined above. The following report, which includes a newly developed clinical scoring system (PNS-Care Score), represents the panel's consensus recommendations.

## Methods

The PNS-Care panel initially consisted of 14 investigators from 8 different countries; all members of the panel are neurologists with clinical and research expertise in PNS and related syndromes. The panel started with the premise that revised consensus diagnostic criteria for PNS were required to improve clinical care and support research. The group established 3 levels of certainty in the diagnosis of PNS (i.e., possible, probable, and definite PNS) according to the coherence between clinical phenotype, antibody, and cancer. In assessing the diagnostic process, the panel reviewed the experience and caveats with detection and interpretation of neuronal antibodies. In addition, new recommendations were considered for neurologic syndromes developing in the context of ICI treatment. It was agreed that several neurologic disorders that can occur in association with cancer are not included in the current diagnostic criteria, such as inflammatory myopathies (dermatomyositis, polymyositis, and necrotizing myopathies), myasthenia gravis, polyneuropathies associated with monoclonal gammopathies, and paraneoplastic retinopathy, optic neuritis, and cochlea-vestibulopathy. Well-designed diagnostic criteria already exist for most of these entities, which are historically not included within the spectrum of PNS.

An initial draft of the guidelines was discussed during the inaugural meeting in Lyon (France) and subsequently underwent several iterations via electronic communication. The last version was then sent to all 14 members, in addition to 5 additional international experts, for final review and comment. All 19 PNS-Care panel members endorsed the final guidelines.

### Data Availability

Data sharing is not applicable to this article as no new data were created or analyzed in this study.

## Results

### Definition of Paraneoplastic Neurologic Syndromes

PNSs are defined as neurologic disorders that (1) can affect any part of the nervous system, often presenting with stereotyped clinical manifestations; (2) occur in association with cancer; and (3) have an immune-mediated pathogenesis that is supported by the frequent presence of specific neuronal antibodies. The 3 parts of this definition represent the main axes of discussion by the panel and constitute the structure of the present guidelines.

### High-Risk Neurologic Phenotypes

There are no absolute pathognomonic neurologic presentations associated with PNS. However, the panel recognizes specific clinical presentations, here defined as “high-risk phenotypes” and previously known as “classical PNS,” frequently have a paraneoplastic etiology. In these phenotypes, cancer represents an important trigger, and therefore, their clinical recognition should lead to a search for an underlying cancer. The extent of cancer search may depend on the demographic characteristics of the patient (age, sex) and the type of neuronal antibody (see below). Despite numerous advances in the field of PNS in the last 16 years, including the discovery of new antibodies and novel clinical manifestations, the panel agrees that there are no new descriptions of high-risk phenotypes, and therefore, the list is as below:

- Encephalomyelitis
- Limbic encephalitis
- Rapidly progressive cerebellar syndrome
- Opsoclonus-myoclonus
- Sensory neuronopathy
- Gastrointestinal pseudo-obstruction (enteric neuropathy)
- Lambert-Eaton myasthenic syndrome

#### Encephalomyelitis

The term encephalomyelitis (EM) should be used only in patients with clinical dysfunction at multiple sites of the nervous system, including also peripheral involvement such as dorsal root ganglia, peripheral nerve or nerve roots, as recommended in the 2004 PNS criteria.^7^ These additional areas of involvement should be included in the description of the phenotype, for example, EM with dorsal root ganglionitis or sensory neuronopathy (SNN) or EM with peripheral neuropathy. EM almost always associates with small-cell lung cancer (SCLC) with Hu (also called antineuronal nuclear antibody 1, ANNA-1) or CV2/collapsin response-mediator protein 5 (CRMP5) antibodies.^8,9^

#### Limbic Encephalitis

Limbic encephalitis (LE) usually presents with short-term memory loss, seizures, and psychiatric manifestations rapidly progressing in less than 3 months. The diagnostic criteria of LE were updated in 2016,^10^ and in this phenotype, the most advances have been made in terms of antibody discovery since 2004. At that time, paraneoplastic and autoimmune LEs were clearly underdiagnosed, and the frequency of reported cases was substantially lower compared with rapidly progressive cerebellar syndromes and sensory neuronopathies.^11^ Importantly, some of the most frequent cell surface antibodies associate with typically nonparaneoplastic forms of LE, such as leucine-rich glioma-inactivated 1 (LGI1) or contactin-associated protein-like 2 (CASPR2) antibodies.^10^ Therefore, the historical concept of LE as a phenotype predominantly associated with cancer has changed dramatically in the last 10 years. ^12,13^ However, because of multiple variants of LE, such as less common forms almost always associated with cancer, this disorder has been retained as a high-risk phenotype. This is important for 2 reasons: first, because the neurologic presentation of paraneoplastic and nonparaneoplastic cases can be undistinguishable, and second, because some of the associated antibodies (e.g., gamma-aminobutyric-acid B receptor [GABA_B_R]^14,15^ and α-amino-3-hydroxy-5-methyl-4-isoxazolepropionic acid receptor [AMPAR]^16,17^) can manifest as paraneoplastic LE in more than 50% of the cases. Although the presence of onconeural antibodies, such as anti-Hu and anti-Ma2, almost always occurs in adults and associate with an underlying cancer, the detection of Hu antibodies in children with LE is exceptionally rare and usually does not associate with cancer.^18^

#### Rapidly Progressive Cerebellar Syndrome

This disorder, previously known as subacute cerebellar degeneration, is characterized by a rapidly progressive cerebellar syndrome, without substantial cerebellar atrophy at early stages of the disease. Cases with hyperacute onset, unilateral onset, or slowly progressive and insidious clinical course mimicking neurodegenerative diseases have also been reported,^19,20^ but in general, the patients rapidly develop a severe and bilateral cerebellar syndrome limiting activities of daily living in less than 3 months. The panel decided to avoid the term paraneoplastic cerebellar degeneration when referring to the clinical syndrome because the presentation of the cases with or without cancer can be indistinguishable. Although gait ataxia may be the main or sole initial feature, truncal and limb involvement later in the course of the disease are needed to define it as rapidly progressive cerebellar syndrome. Extracerebellar dysfunction, predominantly involving brainstem, may accompany the cerebellar features. Isolated cerebellar symptoms are typical of Yo (also known as PCA-1, Purkinje cell antibody 1)^21^ and Tr/delta/notch-like epidermal growth factor-related receptor (DNER) antibodies.^22,23^ Unlike LE, newly identified antibodies for paraneoplastic (and nonparaneoplastic autoimmune) rapidly progressive cerebellar syndrome have been reported only in isolated case reports or small series of patients (table e-1, links.lww.com/NXI/A491). Future research may help to clarify which aspects of cerebellar dysfunction correlate more with specific antibodies.

#### Opsoclonus-Myoclonus Syndrome

Opsoclonus-myoclonus syndrome (OMS) is characterized by involuntary, high-frequency, chaotic multidirectional saccadic movements without intersaccadic pauses, and nonrhythmic action myoclonus, often involving the trunk, limbs, and head. Additional features include cerebellar involvement (dysarthria and trunk ataxia) and encephalopathy (ranging from confusion to coma).

Two main etiologies for OMS include paraneoplastic and idiopathic mechanisms, although there is increasing evidence suggesting that the latter is usually an immune-mediated, postinfectious process. Paraneoplastic OMS in children accounts for 50% of cases and is closely associated with neuroblastoma.^24^ Paraneoplastic OMS in adults frequently associates with SCLC or breast cancer. Patients with breast cancer and paraneoplastic OMS usually have Ri antibodies (also known as ANNA-2).^20,25,26^ Compared with adults with nonparaneoplastic OMS, those with paraneoplastic OMS are more likely to be older, develop encephalopathy, and have a poorer outcome.^25^ In young women, OMS may appear in association with ovarian teratomas without neuronal antibodies.^27^

#### Sensory Neuronopathy

SNN refers to a phenotype caused by involvement of the sensory neurons of the dorsal root ganglia and manifesting with sensory deficits sometimes accompanied by motor symptoms due to additional involvement of motor nerve roots of peripheral nerves. SNN diagnosis (regardless of etiology) should follow previously reported criteria.^28^ The potential causes of SNN are diverse, including Sjögren syndrome or platinum-based chemotherapy, but a paraneoplastic origin should be especially considered if patients have inflammatory CSF or motor involvement.^29^ The terms sensorimotor/sensory neuropathy, polyradiculopathy, or polyradiculoneuropathy should be used when the clinical and electrophysiologic findings indicate additional involvement of the peripheral nerves or nerve roots. The most frequent antibody specificity for SNN is Hu, followed by CV2/CRMP5 and amphiphysin antibodies.^8,30,31^

#### Gastrointestinal Pseudo-obstruction

This term applies to a clinical picture characterized by recurrent episodes of abdominal pain, distension, constipation, and/or vomiting, without evidence of mechanical obstruction.^32^ An abnormal gastric emptying or small bowel manometry confirms the diagnosis. Gastrointestinal pseudo-obstruction is due to a myenteric plexus dysfunction and may occur along with other features of autonomic involvement, SNN, or EM. The identification of Hu antibodies suggests a paraneoplastic origin,^8,32^ whereas antibodies against ganglionic acetylcholine receptor are more frequently seen in nonparaneoplastic cases.^33^

#### Lambert-Eaton Myasthenic Syndrome

Lambert-Eaton myasthenic syndrome (LEMS) is characterized by the progressive development of proximal muscle weakness that usually starts in the lower limbs and follows with involvement of the upper limbs, distal muscles, and finally the ocular and bulbar muscles. About 90% of patients have symptoms of autonomic dysfunction, which is a hallmark of LEMS, including dry mouth, erectile dysfunction, and constipation. In addition to muscle weakness and dysautonomia, patients have decreased or absent muscle reflexes, which improve after repeat exercise or maximal voluntary contraction.^34^ Clinical suspicion must be confirmed with electrophysiologic studies.^35^

Antibodies against P/Q type voltage-gated calcium channels (VGCCs) are present in nearly 90% of the patients, although their detection is not needed for the diagnosis. These antibodies occur similarly in the paraneoplastic and nonparaneoplastic forms of the disease.^34^ Conversely, antiglial nuclear antibodies (or SOX1 antibodies) are strongly associated with SCLC or paraneoplastic syndromes associated with SCLC; therefore, their detection in patients with LEMS strongly suggests the presence of an underlying SCLC.^36^ In addition, the Dutch-English LEMS tumor association prediction score is based on clinical criteria and is useful in the discrimination between paraneoplastic and nonparaneoplastic LEMS.^37^

### Intermediate-Risk Phenotypes

Intermediate-risk phenotypes are neurologic disorders that can occur with or without cancer. The recognition of these phenotypes should prompt consideration of a PNS, particularly when no alternative explanation is found, and patients should be tested for neuronal specific antibodies.

The Panel proposes to consider a possible intermediate-risk phenotype when the onset is rapidly progressive (<3 months) or there are inflammatory findings in the CSF or brain/spine MRI. The panel acknowledges that the list of possible intermediate-risk phenotypes is far from complete but listed below are some of the most suggestive ones:

Encephalitis other than well-defined LE can be considered as intermediate risk phenotype if diagnostic criteria for possible autoimmune encephalitis are fulfilled and antibodies of high or intermediate risk are detected (see below and tables 1 and 2).^10^ This applies especially for those cases with multifocal or diffuse involvement not restricted to the limbic system, such as anti-mGluR5 (metabotropic glutamate receptor 5; associated with Hodgkin lymphoma),^38^ or anti-GABA_A_R encephalitis (gamma-aminobutyric-acid A receptor; associated with malignant thymoma in adult patients).^39^

**Table 1:**
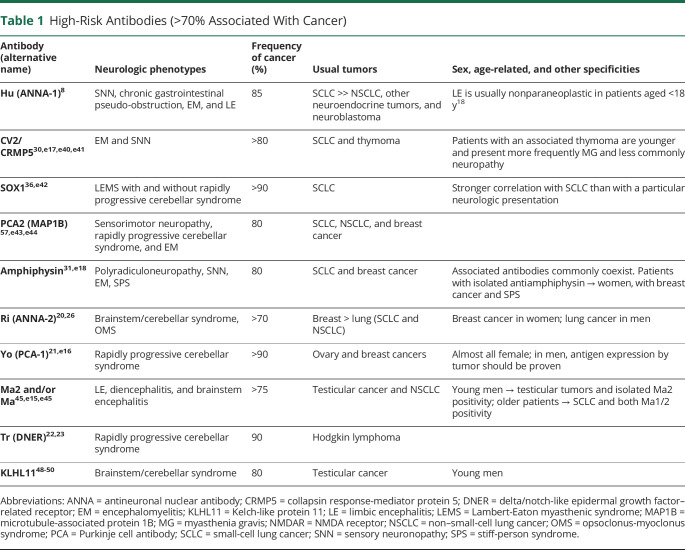
High-Risk Antibodies (>70% Associated With Cancer)

**Table 2:**
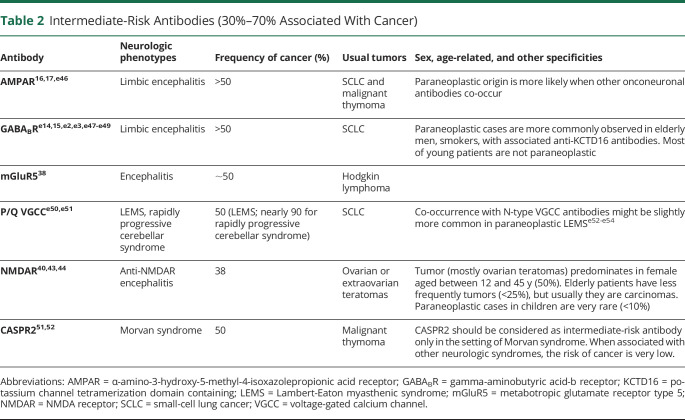
Intermediate-Risk Antibodies (30%–70% Associated With Cancer)

A condition with well-defined diagnostic criteria but unusual oncologic associations is anti-NMDAR encephalitis.^10^ The panel considers this disease as an intermediate-risk phenotype because the presence of an associated tumor highly depends on age and sex.^40^ Children of both sexes and young adult men rarely have tumors but women aged between 18 and 35 years often have an ovarian teratoma, with frequencies ranging between 35% and 50%. In most cases, the teratoma is mature and therefore benign, yet pathologic studies show that they contain NMDAR-expressing neural tissue and often structures that may act as ectopic germinal centers, with tumor-resident NMDAR antibody–producing B cells, directly contributing to the PNS.^41,42^ Immature ovarian teratomas are less common but more frequent than in the general population,^41^ and other malignant tumors occur almost exclusively in elderly patients.^43,44^

Brainstem encephalitis usually presents with oculomotor abnormalities and bulbar symptoms (dysarthria, dysphagia), sometimes accompanied by abnormal movements or cerebellar dysfunction. Brainstem encephalitis may co-occur with LE and is strongly associated with Ma2 antibodies, usually with underlying testicular tumors or non-SCLC (NSCLC).^45^ Diencephalic involvement may accompany brainstem encephalitis in patients with Ma2 antibodies, characterized by excessive daytime sleepiness/narcolepsy, hyperphagia, hyperthermia, and endocrine abnormalities.^45^ The presence of bulbar dysfunction and central hypoventilation is characteristic of Hu antibodies,^46^ whereas OMS and jaw dystonia are common with Ri antibodies.^20,47^ Sensorineural deafness is frequent in brainstem encephalitis associated with KLHL11 antibodies and testicular cancer or teratomas.^48-50^

Morvan syndrome is defined by peripheral nerve hyperexcitability along with encephalopathy characterized by behavioral change, hallucinations, dysautonomia, and sleep disorders, especially agrypnia excitata. Importantly, the co-occurrence of LE and neuromyotonia should not be considered as a synonym of Morvan syndrome. Malignant thymoma is the tumor more commonly associated with Morvan syndrome, frequently accompanied by myasthenia gravis.^51,52^ Morvan syndrome is almost always associated with CASPR2 antibodies, sometimes with concurrent LGI1 and netrin 1 receptor antibodies.^51,52^

Isolated myelopathy as a paraneoplastic manifestation of cancer may have a variable clinical evolution and usually presents with longitudinally extensive, symmetric, tract or gray-matter specific abnormalities in MRI studies. It is mainly associated with breast and lung carcinomas, and with CV2/CRMP5 and amphiphysin antibodies.^53^ However, patients may not have neuronal antibodies, and in these cases, the possibility of a paraneoplastic origin should be considered when MRI is suggestive, and there are no alternative diagnoses.

Stiff-person syndrome (SPS) is characterized by painful muscular spasms that can be spontaneous or triggered by activity or external sensory stimuli and occurs in association with stiffness due to coactivation of agonist and antagonist muscles. Paraneoplastic SPS is mostly associated with amphiphysin antibodies and breast cancer. Compared with the nonparaneoplastic SPS, usually associated with glutamic acid decarboxylase 65 (GAD65) antibodies, patients with amphiphysin-related paraneoplastic SPS are older and frequently have neck and upper limb involvement.^54^ Although some patients with anti–GAD65-associated SPS may have cancer, a paraneoplastic etiology should not be considered unless GAD65 is found expressed by the tumor cells. Besides focal variants of SPS (such as stiff-leg syndrome) that show the same antibody and tumor associations than classic SPS, another disorder lying within the SPS spectrum is progressive encephalomyelitis with rigidity and myoclonus, which usually presents with hyperekplexia, brainstem dysfunction, and dysautonomia, and is related mostly to glycine receptor antibodies in a nonparaneoplastic context.^55,56^

Paraneoplastic polyradiculoneuropathies have typically an axonal pattern and often present with concurrent CNS involvement. Pain, dysautonomia, and distribution (symmetric or asymmetric) are variable. The most frequent antibodies are CV2/CRMP5,^30^ amphiphysin,^31^ and PCA-2/microtubule-associated protein 1B,^57^ usually in the context of SCLC, or breast cancer also in association with amphiphysin antibodies. In patients with cancer, the development of neuropathies that fulfill the criteria of Guillain-Barré syndrome or chronic inflammatory demyelinating polyneuropathy should not be considered paraneoplastic unless a high-risk antibody is identified (table 1).

### Cancer Associated With PNS and Cancer Screening

The panel agreed that the demonstration of a causal, not coincidental, association between the underlying tumor and the neurologic phenotype is crucial for the definite diagnosis of PNS. Although some progress has been made in the characterization of this pathogenic link, such as the identification of specific mutations or amplifications in the genes encoding for onconeural antigens in tumors of patients with PNS,^58^ in clinical practice, this link is suggested by:

#### Epidemiologic Associations

Clinical series indicate that distinct types of PNS preferentially associate with certain types of cancers, regardless of the presence or absence of antibodies, and type of antibody. For example, rapidly progressive cerebellar syndrome in postmenopausal women is frequently paraneoplastic, and the tumors more frequently involved are breast and ovarian cancer (in this case, patients usually have Yo antibodies). Another example is OMS in children with neuroblastoma (in this case, patients do not have a specific antibody).

#### Antibody Associations

Antibodies are important to guide the search for an underlying tumor. In the context of PNS, 3 groups of antibodies can be considered according to the frequency of cancer association regardless of their eventual pathogenic effect. The first group includes antibodies that occur very frequently (>70%) in patients with an underlying cancer (table 1). In the 2004 PNS criteria, these antibodies were defined as onconeural antibodies to emphasize the link between cancer and brain. However, it is now clear that some antibodies associate less frequently with cancer, for example, AMPAR and NMDAR, and the target antigens are expressed in both the neurons and the tumor. On the other hand, some of the antigens of classical onconeural antibodies (such as Tr/DNER) are not expressed in the associated tumor (Hodgkin lymphoma). For this reason, the panel proposes to substitute the term onconeural, which implies the obligatory expression of the antigen by the nervous system and cancer, for the term high risk. Most high-risk antibodies target intracellular antigens and are currently considered not to be directly pathogenic but only good biomarkers of PNS. The second group of antibodies occur in association with cancer in 30%–70% of cases (table 2). Finally, the third group of antibodies have a much lower (<30%), or absent, association with cancer (table 3). In cases of PNS without antibodies, the involvement of a tumor is more difficult to demonstrate as it may be coincidental and not pathogenically linked. The tumors more frequently associated with PNS irrespective of the antibody status are SCLC, breast cancer, ovarian cancer, NSCLC, and lymphomas.^13,59,60,e1^

**Table 3:**
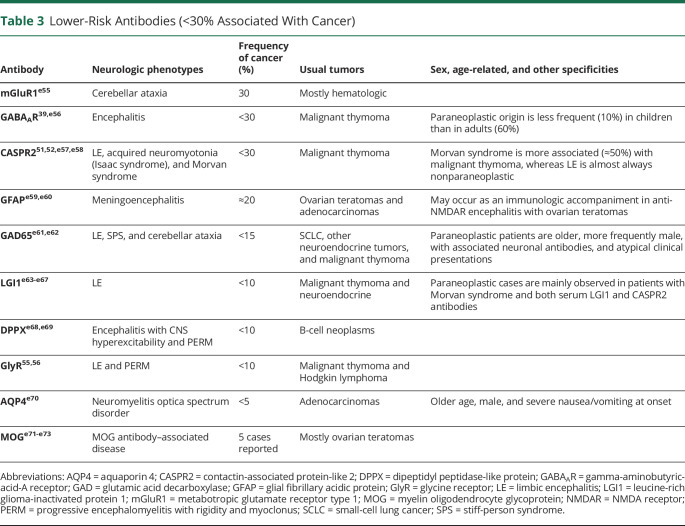
Lower-Risk Antibodies (<30% Associated With Cancer)

In clinical practice, the indicated antibody associations with cancer have important clinical implications. For a specific phenotype, for example, LE, the presence of one antibody vs another suggests the likelihood of having a tumor or not and directs the tumor search. For example, patients with LE and LGI1 antibodies rarely have a tumor, whereas at least 50% of patients with GABA_B_R antibodies have SCLC,^14,15^ more than 50% of patients with AMPAR antibodies have thymoma, lung, or breast cancer,^16,17^ and most (>85%) patients with Hu antibodies have SCLC.^8^

These antibody-tumor associations can show age and sex dependence; for example, NMDAR antibodies rarely associate with tumors in young children or young adult male patients.^40^ Very rarely, Hu^18^ or GABA_B_R^e2,^ ^e3^ antibodies can be identified in children with epileptic encephalopathy, and these patients almost never have cancer.

#### Expression of Neural Antigens by the Tumor

In some clinical research settings (e.g., unexpected tumor and antibody concurrence), the demonstration that the tumor expresses the cognate antigen is critical to pathogenically associate it with the PNS (i.e., Yo antibodies in a man with gastric adenocarcinoma).^e4^ Similar studies are needed when there is limited experience with the PNS or associated antibody (i.e., mGluR2 antibodies and rapidly progressive cerebellar syndrome with sarcoma).^e5^

Cancer screening should be promptly undertaken when a PNS is suspected and should be guided by the type of phenotype or antibody (tables 1 and 2). Patients can have more than 1 tumor; thus, if the identified tumor is atypical for the type of suspected phenotype or antibody, additional studies for a second tumor should be considered.^e6^ Recommendations according to the type of suspected tumor are shown in table e-2 (links.lww.com/NXI/A491).^21,e6-e16^

When initial tumor screening is negative, it should be repeated every 4–6 months for 2 years in patients with high-risk phenotypes along with high-risk antibodies (table 1). The panel decided to establish this time frame based on the members' clinical experience and the evidence from the literature showing that a vast majority of the tumors are diagnosed within 2 years after PNS onset^8,21-23,26,30,31,45,48,e15-e18^; however, this is a general recommendation that should be adapted to every individual case according to risk factors, clinical evolution, and medical resources. The same applies to patients with high-risk phenotypes along with intermediate risk antibodies (table 2) who show particular demographic characteristics (older age and smoking) or have concurrent antibodies with strong cancer association (e.g., P/Q VGCC and SOX1 antibodies in LEMS). For patients who do not fulfill these criteria, and those with lower risk antibodies (table 3), a comprehensive screening for cancer by the time of initial diagnostic assessment is sufficient. Tumor rescreening could be considered in some clinical settings, such as patients refractory to treatment or with relapsing neurologic diseases (e.g., anti-NMDAR encephalitis).

### Neuronal Antibodies as Biomarkers in PNS

Although PNS can be diagnosed without neuronal antibody testing (e.g., pediatric OMS and neuroblastoma; LEMS and SCLC), the demonstration of neuronal antibodies is of extraordinary help in the diagnosis of PNS, and these antibodies have become very important biomarkers of PNS. Gold standard detection methods include rodent brain tissue immunohistochemistry/immunofluorescence (IHC/IF) accompanied by confirmatory studies using immunoblot with recombinant proteins (for most antibodies directed to intracellular antigens) or cell-based assays (CBAs, for antibodies against cell surface or synaptic proteins).^e19,e20^ Brain immunohistochemistry is not useful for 2 antibodies (P/Q type VGCC, glycine receptor antibodies), and the utility of tissue immunohistochemistry is unclear (pending to be better defined) for SOX1 antibody. Recommendations for antibody testing are shown in table 4.

**Table 4:**
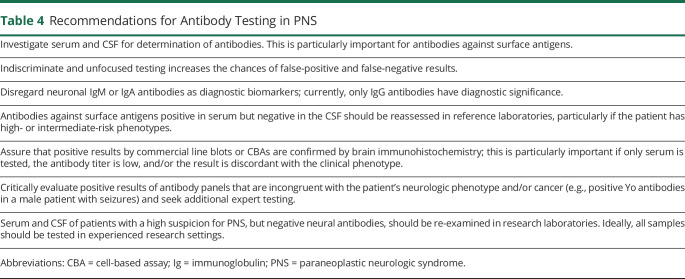
Recommendations for Antibody Testing in PNS

Sensitivity and specificity for serum or CSF analysis vary among different antibodies; it is therefore recommended to perform antibody testing in both samples. Laboratory studies using CBA with serum only have similar problems of false-positive and -negative results. For all suspected autoimmune or paraneoplastic encephalitis associated with antibodies against neuronal surface antigens, screening of CSF should be obligatory to avoid mistakes. Patients with neuronal surface antibodies detected in serum only (CSF negative) should be re-examined in a research laboratories or with confirmatory tissue IHC/IF before considering a definite diagnosis. On the other hand, some antibodies (e.g., against LGI1) are best detected in serum, with CSF showing lower sensitivity. Despite the indicated gold standard techniques mentioned above (brain IHC/IF and CBA), very few laboratories use both techniques.

Commercial kits that test multiple antibodies may be helpful. However, often, the kits detect antibodies of limited clinical value for diagnosis of PNS along with antibodies with well-known clinical and cancer associations that have been validated across different specialized centers. Although commercial line blot kits for antibody detection are useful for the initial screening of patients, the number of false-positive and -negative results is particularly high for line blots assessing Yo, Ma2, CV2/CRMP5, and SOX1 antibodies.^e21-e25^ Unexpected antibody results based on the type of neurologic phenotype, tumor, or patient's age and sex should raise concern for false-positive results and be reassessed with additional studies, preferably in research laboratories. Similarly, reference laboratories should perform the antibody testing in patients with high suspicion for PNS but negative routine screening of antibodies in clinical or commercial laboratories.^e21,e22^

Several antibodies, mostly related to rapidly progressive cerebellar syndrome, are not well characterized yet, because they have been described recently, in small series or isolated case reports, or there is limited experience across different research laboratories (table e-1, links.lww.com/NXI/A491). Further studies involving larger series are needed to confirm the clinical and oncologic associations of these new antibodies and require input of research laboratories to forward the accuracy of patient diagnoses.

### Diagnostic Criteria for PNS

The diagnosis of PNS requires the reasonable exclusion of alternative causes that sometimes are much more prevalent. The differential diagnosis of PNS is wide, as it includes infections, autoimmune nonparaneoplastic diseases, tumors, neurodegenerative disorders, and toxic/metabolic disturbances. Most of these alternative diagnoses are epidemiologically more frequent than PNS, and some of them are treatable; therefore, there is an important need to readily identify them. The differential diagnosis should be based on the clinical presentation and patient's demographic features (table e-3, links.lww.com/NXI/A491). After that, 3 levels of diagnostic certainty are proposed (possible, probable, and definite PNS) based on a scoring system (PNS-Care Score) that considers the type of clinical phenotype, presence or absence of neuronal antibodies, and presence or absence of cancer (table 5).

**Table 5:**
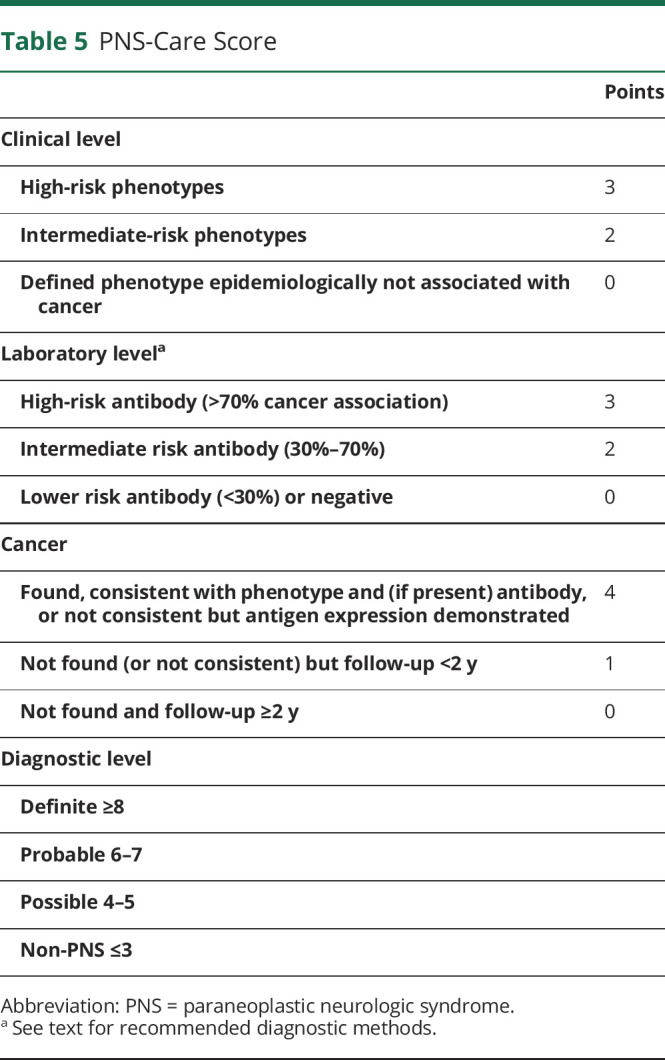
PNS-Care Score

The panel recognizes that the proposed criteria may underestimate the occurrence of cases of PNS without neuronal antibodies, but the use of these biomarkers provides unambiguous diagnostic certainty and enables to homogenize samples for research purposes. According to these criteria, the diagnosis of definite PNS (score ≥8) includes the presence of a high- or intermediate-risk phenotype (as previously described) along with a high- or intermediate-risk antibody, and the presence of cancer. The presence of cancer is mandatory to define definite PNS. If the cancer is unusual for the type of antibody found, the diagnosis of definite PNS requires the demonstration of antigen expression by the tumor.

The panel proposed as exception the OMS associated with neuroblastoma or SCLC in which there is no specific antibody association. Therefore, although this syndrome provides a score of 7, it should be considered definite PNS when associated with these tumors. The panel also acknowledges that the present criteria do not identify as definite PNS neurologic syndromes associated with cancer and low-risk antibodies even if tumor cells express the neuronal antigen recognized by the antibody (e.g, neuromyelitis optica with aquaporin 4 antibodies and concurrent lung adenocarcinoma that expresses aquaporin 4).

Note that the diagnostic level of probable or possible PNS (table 5) may change over time according to the length of follow-up, greater or less than 2 years. For example, a patient with LEMS, VGCC antibodies but no cancer at diagnosis has a score of 6 (probable). If an SCLC is found 18 months later, the diagnosis will be upgraded to definite (score 9), but if no cancer is found after >2 years, the diagnosis will be downgraded to possible (score 5).

### The Era of Immune Checkpoint Inhibitors

ICIs enhance antitumor immunity by blocking immune checkpoint molecules expressed in T lymphocytes and tumor cells including programmed cell death protein 1, its ligand, and cytotoxic T-lymphocyte–associated antigen 4.^e26^ The adoption of ICI treatment in oncologic practice has led to increased survival and long-term remissions, even in patients with extensive metastatic cancer.^e26^ The major downside of ICIs is the possibility of developing immune-related adverse events (irAEs),^e27^ including severe (grade 3 or higher) neurologic syndromes (1%–3% of the cases).^5,e28^ These include the worsening of preexisting and de novo development of autoimmune neurologic diseases. The panel recommends that the first step in approaching these disorders is to determine whether the syndrome fulfills the above-mentioned criteria for PNS, after having excluded other alternative etiologies (e.g., carcinomatous meningitis).^e29^ Both peripheral and CNS complications have been described.^e30,e31^ There is already evidence that specific neurologic syndromes (e.g., those associated with Ma2 and Hu antibodies) can be triggered by cancer immunotherapy.^6,e32^ Nevertheless, a substantial proportion of cases remain seronegative despite comprehensive screening, and the detection of antibodies is not required for the diagnosis of irAEs. Although classical PNSs are known to precede the discovery of cancer, neurologic syndromes triggered by ICIs by definition develop when the cancer is already diagnosed, in general shortly after the initiation of ICIs.

Of interest, for the few patients who developed antibody-associated irAEs and samples taken before ICI introduction were available, the retrospective analysis revealed the presence of Ma2 or Hu antibodies before the onset of PNS in 4 cases,^6,e33-e35^ similarly to what it was observed in 3 cases of ICI-triggered myasthenia gravis.^e36-e38^

The optimal management of ICI-induced neurologic autoimmunity has not been established and is beyond the scope of this diagnostic guidelines, but the panel recommended the following considerations: (1) neuronal antibody testing needs to be routinely performed in all patients developing neurologic irAEs resembling high or intermediate risk PNS; (2) patients with current or previous PNS are at a higher risk of developing neurologic worsening if treated with ICIs, and therefore, the risk/benefit ratio of ICI should be carefully weighted in this setting. For example, 50% of cases with preexistent PNS worsened during ICI treatment in a recent study^e39^; (3) future studies should address the potential value of assessing neuronal antibodies before starting ICIs, particularly in patients harboring cancers with tendency to associate with PNS (e.g., lung, breast, and ovary cancer); (4) close neurologic follow-up of antibody-positive cases is recommended.

#### Final Comments

The evaluation of suspected PNS and their management requires detailed (and evolving) knowledge, so as to permit timely and accurate diagnosis of these uncommon disorders. Unambiguous diagnostic criteria facilitate both timely diagnosis (which may affect the neurologic and oncologic outcome) and avoidance of overdiagnosis and unnecessary treatments. In addition, these criteria represent an important research tool for epidemiologic studies and to analyze the value of new antibodies for the diagnosis of PNS. For the reasons stated in the introduction, modification of the 2004 criteria was necessary to accommodate the new knowledge generated in the last 16 years. The update criteria presented here (1) include novel phenotypes and immune-mediated pathogenic mechanisms identified since 2004; (2) emphasize a causal (and not merely chronological) association with cancer; and (3) require the demonstration of neuronal antibodies using gold standard techniques. These 3 elements represent the core of the present criteria of PNS that we hope will be of help to clinicians and researchers.

## Glossary

AMPAR: α-amino-3-hydroxy-5-methyl-4-isoxazolepropionic acid receptor
ANNA: antineuronal nuclear antibody
CASPR2: contactin-associated protein-like 2
CBA: cell-based assay
CRMP5: collapsin response-mediator protein 5
DNER: delta/notch-like epidermal growth factor-related receptor
EM: encephalomyelitis
GABA_B_R: gamma-aminobutyric-acid B receptor
GAD65: glutamic acid decarboxylase 65
ICI: immune checkpoint inhibitor
IHC/IF: immunohistochemistry/immunofluorescence
irAE: immune-related adverse event
LE: limbic encephalitis
LEMS: Lambert-Eaton myasthenic syndrome
LGI1: leucine-rich glioma-inactivated 1
mGluR: metabotropic glutamate receptor
NSCLC: non-SCLC
OMS: opsoclonus-myoclonus syndrome
PCA: Purkinje cell antibody
PNS: paraneoplastic neurologic syndrome
SCLC: small-cell lung cancer
SNN: sensory neuronopathy
SPS: stiff-person syndrome
VGCC: voltage-gated calcium channel
